# A Paradigm Shift in the Diagnosis of Aspiration Pneumonia in Older Adults

**DOI:** 10.3390/jcm11175214

**Published:** 2022-09-03

**Authors:** Yuki Yoshimatsu, David G. Smithard

**Affiliations:** 1 Elderly Care, Queen Elizabeth Hospital, Lewisham and Greenwich NHS Trust, London SE18 4QH, UK; 2 Centre for Exercise Activity and Rehabilitation, School of Human Sciences, University of Greenwich, London SE10 9LS, UK

**Keywords:** dysphagia, swallowing disorder, aspiration, diagnosis, differential, community-acquired pneumonia, CAP, frailty

## Abstract

In older adults, community-acquired pneumonia (CAP) is often aspiration-related. However, as aspiration pneumonia (AP) lacks clear diagnostic criteria, the reported prevalence and clinical management vary greatly. We investigated what clinical factors appeared to influence the diagnosis of AP and non-AP in a clinical setting and reconsidered a more clinically relevant approach. Medical records of patients aged ≥75 years admitted with CAP were reviewed retrospectively. A total of 803 patients (134 APs and 669 non-APs) were included. The AP group had significantly higher rates of frailty, had higher SARC-F scores, resided in institutions, had neurologic conditions, previous pneumonia diagnoses, known dysphagia, and were more likely to present with vomiting or coughing on food. Nil by mouth orders, speech therapist referrals, and broad-spectrum antibiotics were significantly more common, while computed tomography scans and blood cultures were rarely performed; alternative diagnoses, such as cancer and pulmonary embolism, were detected significantly less. AP is diagnosed more commonly in frail patients, while aspiration is the underlying aetiology in most types of pneumonia. A presumptive diagnosis of AP may deny patients necessary investigation and management. We suggest a paradigm shift in the way we approach older patients with CAP; rather than trying to differentiate AP and non-AP, it would be more clinically relevant to recognise all pneumonia as just pneumonia, and assess their swallowing functions, causative organisms, and investigate alternative diagnoses or underlying causes of dysphagia. This will enable appropriate clinical management.

## 1. Introduction

In this unprecedented ageing world, clinicians are facing the increasing impacts of community-acquired pneumonia (CAP) in older adults on a regular basis. Its high prevalence and morbidity are causing large medical burdens, while also imposing socioeconomic consequences on the whole of society [1]. The incidence of pneumonia has increased from 1.5 to 2.2 per 1000 person years between 2002 and 2017 [2], with the prevalence being 6 times higher in those aged ≥75 years old compared to those <60 years of age [3]. CAP has a mortality of 2–5/1000 years [4,5], and in 2019 there were 2.5 million deaths from pneumonia [6].

Aspiration is considered to be the likely aetiology of pneumonia in up to 90% of older adults [7], where dysphagia is common [8]. Consequently, nearly all pneumonia will be associated with the aspiration of oropharyngeal secretions. However, swallowing problems alone may not be the cause of aspiration pneumonia (AP) [9], and the clinical effects may depend on the frailty of the patient, the oral microbiome load due to insufficient oral care, and a decompensated airway clearance [10,11]. Having an adequate cough reflex, the urge to cough, and the necessary pulmonary function are all important aspects in the prevention of pneumonia. Sarcopenia is also known to be a risk factor for pneumonia in older people [12]. Many people will have a pre-existing compromised swallow, a previously unidentified swallowing problem, or a decompensated swallow due to illness and frailty. Patients with a diagnosis of AP are at risk of longer and more frequent admissions, are more likely to be frail, and have multiple comorbidities [13,14]. Therefore, their clinical management requires multi-professional assessment and care, in addition to those for CAP in general.

There are no clear diagnostic criteria for AP, and the diagnosis is often presumptive [7]. British and American pneumonia guidelines do not state a definition [15,16]; Japanese guidelines merely state a list of risk factors for aspiration and pneumonia, explaining that clear criteria cannot be easily established [17]. Understandably, this implies an inconsistency in what clinicians and researchers infer when they mention the term “AP”. Its reported ratio also varies greatly, ranging from 5.6% to as high as 90% among those diagnosed with CAP [7,13,18,19,20]. A need for clarity in the diagnosis of AP has been voiced for decades. Though some researchers have made suggestions [21], there are yet to be unified criteria.

Subsequent to the absence of a unified definition of AP, the management of pneumonia in older adults remains variable [22]. When a diagnosis of AP is made, it is common to restrict patients’ oral intake. When a patient has a repetitive diagnosis of AP, it leads clinicians to suspect entry of food and liquids into the airway as being the cause, and often to the decision of nil by mouth (NBM), while the need for (artificial) nutrition is ignored. However, how can clinicians make such crucial decisions based on such unreliable diagnoses? With the prognosis of AP being poor [22], adequate management is crucial.

Though there have been increasing numbers of studies suggesting how a diagnosis of AP is reached [21,23], the appropriateness of the diagnosis or how AP is actually being diagnosed in the daily clinical setting remains largely unexplored. This is especially so in the UK, as many studies originate from Japan, Spain, and elsewhere [23]. Without real-world data, it is impossible to know the real issues that need to be addressed. Therefore, we designed a study to investigate what clinical factors appear to influence the diagnosis of AP, and how the differentiation affects its management in the acute geriatric setting. This study was performed to reconsider a clinically relevant way to diagnose AP in older adults.

## 2. Materials and Methods

### 2.1. Study Design

This was a retrospective cohort study analysing how older patients admitted with CAP were diagnosed as having AP or non-AP, and whether their management differed depending on the diagnosis. The study was performed at Queen Elizabeth Hospital (Lewisham and Greenwich NHS Trust), a local 521-bed acute care hospital serving the south-eastern London area of England. Ethical approval was provided by the Lewisham and Greenwich NHS Trust as instituted by the Declaration of Helsinki, and informed consent was waived.

### 2.2. Patients

Patients aged ≥75 years admitted with a diagnosis of CAP from the emergency department from 1 January to 31 December, 2021 were included in the study. A list of patients with pneumonia or pneumonitis as a primary or secondary diagnosis of admission was obtained from the hospital database. Exclusion criteria were admissions for hospital-acquired pneumonia (HAP), cases that were diagnosed with pneumonia after initially being admitted for a different condition, COVID-19 pneumonitis, and cases without pneumonia according to medical records (a discrepancy between the coded database and written medical records). Patients diagnosed with infectious exacerbation of chronic obstructive pulmonary disease (COPD) were included if they were also diagnosed with pneumonia. Patients who were admitted multiple times during the study period were only included for the first admission, and any admissions thereafter were excluded.

### 2.3. Definitions

CAP was defined as pneumonia developing in the community or within 48 h of hospital admission [15], and HAP was defined as pneumonia not incubating at the time of hospital admission and occurring 48 h or more after admission [24], according to the American Thoracic Society (ATS) and the Infectious Diseases Society of America (IDSA) guidelines. The diagnoses of pneumonia and AP were extracted according to what was documented on the consultant physician ward round at the time of admission. This was because our study intended to investigate the reality of how AP was being diagnosed in the everyday clinical setting. Non-AP was defined as any patient diagnosed with CAP that was not documented as AP.

### 2.4. Data Collection

The following data were collected from medical records: patient demographics (age, sex), social history (whether they lived at home or in a care/nursing home), medical history (comorbidities, drugs, pneumonia within the past year, risk factors for multi-drug resistant organisms in pneumonia and healthcare-associated pneumonia (HCAP) [25,26]), presenting condition (CURB-65 score [27], pneumonia severity index [28], clinical frailty score [29], SARC-F score [30]), initial investigations undertaken in the emergency department (chest X-ray, chest computed tomography (CT), blood culture, sputum culture, urine pneumococcal and legionella antigen), initial diagnosis (AP or non-AP), other additional diagnoses, and management (initial antimicrobial treatment, initial NBM orders, speech and language therapist (SLT) referral, videofluoroscopic swallow study (VFSS), and fibreoptic endoscopic evaluation of swallowing (FEES)).

### 2.5. Antimicrobial Treatment

In the UK, each National Health Service (NHS) Trust has its own antimicrobial guidelines, depending on the drug sensitivity of the local microbiome. The guidelines are expected to be followed unless recommended otherwise. At the Lewisham and Greenwich NHS Trust (in which this study was performed), the recommended microbial treatment for pneumonia and lung infection is as follows:

1. CAP: amoxicillin, or amoxicillin and clarithromycin, depending on CURB-65 score (if allergic to penicillin: vancomycin and clarithromycin).
2. AP: amoxicillin, metronidazole, and gentamicin (if allergic to penicillin: teicoplanin, metronidazole, and gentamicin).
3. HAP (including CAP presenting within 1 month of discharge from hospital): amoxicillin and gentamycin, or amoxicillin and clavulanic acid, or amoxicillin, clavulanic acid, and amikacin (if allergic to penicillin: teicoplanin and gentamicin).
4. Infectious exacerbation of COPD: doxycycline.

Medical records were checked to see what antimicrobials were used, and whether the initial treatment consisted of the triple therapy for AP or not.

### 2.6. Statistical Analyses

Patients were separated into two groups according to the initial diagnosis: AP or non-AP. Patient background, symptoms, and management were compared between the two groups. Descriptive statistics for baseline data were presented as the percentage, median, and interquartile range. The Mann–Whitney U-test was used for continuous variables, and the chi-square test or Fisher’s exact test for categorical variables. All data analyses were carried out using Microsoft Excel (2018, Microsoft Corporation, Redmond, WA, USA) and Social Science Statistics, 2022 [31,32]. A *p* value < 0.05 was considered to be statistically significant.

## 3. Results

### 3.1. Patient Selection

**Figure 1:**
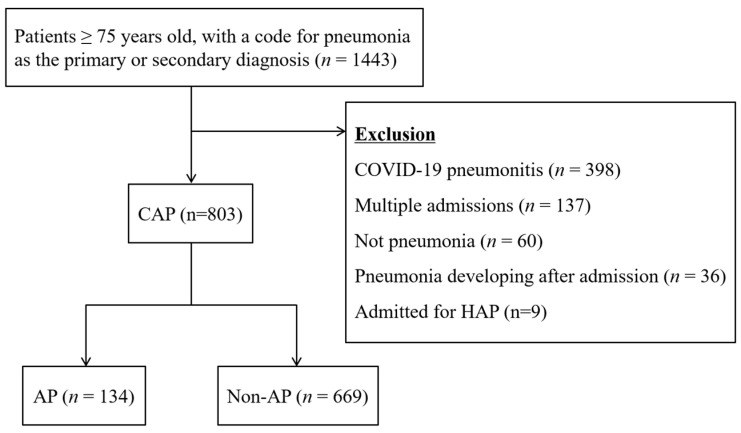
The patient selection process. A total of 1443 patients were listed as having a primary or secondary diagnosis of pneumonia or pneumonitis. According to the exclusion criteria, 640 cases were excluded. This included 398 cases with COVID-19 pneumonitis, 137 with multiple admissions in the study period, 60 with no pneumonia according to medical records, 36 who had developed pneumonia after the admission, and 9 who were admitted for hospital-acquired pneumonia (HAP). As a result, 803 cases of community-acquired pneumonia (CAP) were included in the study. Of the 803 cases, 134 were initially diagnosed as having aspiration pneumonia (AP group), and the remaining 669 cases constituted the non-AP group.

The patient selection process is shown in Figure 1. On the hospital database, a total of 1443 patients were listed as admitted with a primary or secondary diagnosis of pneumonia or pneumonitis during the study period. According to the exclusion criteria, 640 cases were excluded. This included 398 cases with COVID-19 pneumonitis, 137 with multiple admissions in the study period, 60 with no pneumonia according to medical records, 36 who had developed pneumonia after the admission, and 9 who were admitted for HAP. As a result, 803 cases were included in the study. Of the 803 cases, 134 (16.7%) were initially diagnosed as having aspiration pneumonia (AP group), and the remaining 669 cases constituted the non-AP group.

### 3.2. Patient Background

The patient demographic data are shown in Table 1. In total, there were 423 males (52.7%), and the median age was 84 years old (interquartile range: 80–89). There was no significant difference in the patient sex and age between the two groups. It was significantly more common for patients from the AP group to come from a care home or nursing home than in the non-AP group (29.9% vs. 11.3%, respectively, *p* < 0.05). Regarding their general well-being, the AP group had a significantly higher clinical frailty score (median 6 vs. 7) and SARC-F score (median 7 vs. 4) than in the non-AP group (*p* < 0.05). The patient demographic data excluding the 51 cases in which there was no detectable pneumonia on a CT scan (explained in 3.7) are shown in the Appendix A (Table A1). After exclusion, in the AP group, the comorbidities of ischemic/congestive cardiac conditions and type 2 diabetes mellitus were seen significantly more frequently in the AP group (*p* < 0.05), while having a history of pneumonia within 1 year of admission became non-significant (*p* = 0.06).

### 3.3. Past Medical History

Data on past medical history are also shown in Table 1. Regarding comorbidities, those in the AP group were significantly more likely to have a history of neurologic conditions and dementia, and less likely to have a chronic respiratory disorder (*p* < 0.05). There was no significant difference between the two groups regarding the prevalence of stroke, other mental disorders (such as schizophrenia, depression, and epilepsy), gastroesophageal reflux disease (GORD), other gastroesophageal disorders, cardiac conditions, type 2 diabetes mellitus, active cancer, or head and neck cancer. As for risks of HCAP, the AP group had a significantly higher rate of having had pneumonia in the past year, a recent admission within 90 days (*p* < 0.05), and a significantly lower rate of immunodeficiency (*p* < 0.05) (haematologic condition, chemotherapy, or systemic steroids). There was no significant difference in the number of regular drugs, rate of haemodialysis, or having intravenous antibiotics/chemotherapy/wound care within 30 days. The AP group had a significantly higher rate of having known dysphagia (44.8% vs. 30.0%, *p* < 0.05).

### 3.4. Symptoms and Signs

Symptoms and signs at admission are shown in Table 2. There were significantly fewer patients in the AP group that complained of pleuritic pain, dyspnoea, and fever than in the non-AP group. There were significantly more patients in the AP group that had a history of symptoms suggestive of an aspiration: altered mental status, coughing on food, and vomiting. There were no significant differences in the two groups regarding cough or purulent sputum. No patient was assessed for their swallow before being diagnosed as AP or non-AP. With regard to the severity of pneumonia, the AP group had higher scores of CURB-65 and PSI than in the non-AP group (*p* < 0.001). The signs and symptoms of the patients, excluding the 51 cases in which there was no detectable pneumonia on a CT scan (explained in 3.7), are shown in the Appendix A (Table A2). There were no significant differences between the two tables. 

### 3.5. Diagnostic Investigations

The diagnostic investigations performed on admission regarding the pneumonia are shown in Table 3. In the AP group, blood culture, urine Legionella antigen tests, or chest CTs were significantly less likely to be performed than in the non-AP group. In both groups, sputum cultures and urine *S. pneumoniae* antigen tests were rarely performed, showing no significant difference in the frequency.

### 3.6. Alternative Diagnoses of CT

Among the 803 cases initially diagnosed with pneumonia, 130 underwent a CT scan upon the orders of the emergency physician or consultant in charge of admission. In most cases, the CTs were performed in order to rule out other diagnoses, such as pulmonary embolism or aortic dissection, or to investigate a suspected lung tumour. The scan was carried out within the following few days, and the results are shown in Table 4. In 51 of the 130 cases (39.2%), there was no pneumonia detected on the CT scans. In 56 cases (43.1%), there was another diagnosis found on CT, such as a new cancer (13.1%), new lung metastases of a known cancer (6.2%), new lung nodules (2.3%), pleural effusion (4.6%), pulmonary oedema (2.3%), and others, such as interstitial lung disease and pneumothorax.

### 3.7. Diagnosis of New Causative Conditions of Dysphagia and Aspiration

In a total of 35 cases (4.4%), a cause of dysphagia or aspiration not detected on admission was newly diagnosed later during the admission (Table 5). This constituted 13 cases (9.7%) from the AP group, and 22 cases (3.3%) from the non-AP group. The most commonly found were neurologic conditions (37.1%), including acute stroke and dementia. The second common cause was gastrointestinal conditions (28.6), including hiatal hernia and metastatic oesophageal obstruction. This was followed by drug-induced aspiration (17.1%), such as vomiting and altered mental status caused by hypercalcemia from osteoporosis treatment, or hypo-delirium due to antipsychotics or antidepressants. Other causes included head and neck conditions and cardiopulmonary conditions.

### 3.8. Management

The treatment and interventions are also shown in Table 3. Antimicrobial treatment was initiated in all patients except for one patient in the non-AP group who was recognised to be at the end of life and managed with palliative care. Antimicrobial regimens for AP were selected as the initial treatment significantly more often in the AP group than in the non-AP group (53.0% vs. 2.8%, respectively, *p* < 0.05).

In the AP group, NBM orders and speech and language therapist referrals were significantly more common than in the non-AP group (*p* < 0.05). VFSS/FEES were only performed in seven cases in total.

## 4. Discussion

We conducted a retrospective study to investigate factors utilised to differentiate AP from non-AP, and the consequential management of the two groups. Statistically significant differences were found between the groups, in factors leading to the diagnosis and their management, giving us an idea of how a diagnosis of AP is made and acted upon.

### 4.1. Diagnosis of Aspiration Pneumonia

Results showed that patients who were more likely to be diagnosed with AP than non-AP were those with the following factors: residing in care/ nursing homes, a history of neurologic conditions or dementia, previously diagnosed dysphagia, recent pneumonia, recent admission, frailer, and have a higher SARC-F score. As for symptoms, patients diagnosed with AP were significantly more likely to present with altered mental status, vomiting, or coughing on oral intake. No swallow function screenings or assessments were performed by the physicians diagnosing the pneumonia; no evaluation of airway clearance was taken into account. There were no cases in which diagnoses were postponed until after the SLT assessment. These findings suggest that clinicians may be more inclined to diagnose AP in a generally frail patient with more comorbidities who have symptoms suggestive of an aspiration than on the basis of a swallowing disorder. While these are common factors of AP, they are neither directly indicative of aspiration nor are they sufficient to make definitive diagnoses of AP. Assessments of the swallow and cough effectiveness are recommended [11]. Furthermore, vomiting imposes patients to aspiration pneumonitis, which is a chemical reaction of the lungs to the acidic aspirates of vomitus and differs from AP in diagnosis and management. In clinical practice, efforts to distinguish between the two may be neglected and antibiotics given, as it may seem to be the choice with less immediate risk.

On the contrary, patients with a coexisting chronic respiratory disorder were significantly less likely to be diagnosed as AP. These included COPD, interstitial lung disease, and bronchiectasis. When clinicians see patients with these coexisting respiratory conditions, they may tend to diagnose them with ‘infective exacerbation of the underlying condition’ and may be less likely to suspect aspiration as an additional underlying cause. However, it is known that chronic respiratory conditions [33], especially COPD [34], predispose patients to dysphagia. Moreover, aspiration can be the cause of infective exacerbations of COPD [35,36], exacerbations of interstitial lung disease [37,38], and adult bronchiectasis [39,40,41]. Therefore, a coexisting respiratory condition or an infective exacerbation should not discourage clinicians from suspecting aspiration; rather, it should prompt attention towards assessing the swallowing function and the possibility of the underlying aspiration.

### 4.2. Microbial Investigations

Following the diagnosis of AP or non-AP, further investigations were ordered by the emergency department, including blood and sputum cultures, and urine pneumococcal and legionella antigen tests. Blood cultures and urine legionella antigen tests were performed significantly less frequently in the AP group than the non-AP group. In any pneumonia, efforts to identify the causative organism and rule out other possible conditions are important for treatment. A diagnosis of AP or a state of frailty is not a reason against the microbial investigation. If anything, patients with AP, who often fall under the group of HCAP and are prone to resistant organisms [42], in addition to oral streptococcus species, require even more attention to identifying the causative organism. This is necessary, not only for the treatment of the presenting pneumonia but also for reference in the event of future infections and to prevent antimicrobial resistance or the nosocomial spread of infection.

Sputum culture was performed particularly infrequently in both groups. There may be a few reasons. In the UK, clinical practice is guided by the National Institute for Health and Care Excellence (NICE) guidelines [43], in addition to specific guidelines by each society. For CAP in adults treated in the hospital, the British Thoracic Society (BTS) guidelines state that ‘sputum samples should be sent for culture and sensitivity tests from patients with CAP of moderate severity who are able to expectorate purulent samples and have not received prior antibiotic therapy’ [16]. The NICE guidelines [43] and the *British Medical Journal* (BMJ) best practice guidelines [44] are also in line with this recommendation. It is suspected that a substantial proportion of these patients could not expectorate purulent samples initially or had received prior antibiotic therapy. Further, the COVID-19 pandemic has made it difficult to collect sputum in open environments such as emergency departments or normal wards. Nonetheless, sputum culture should be considered in the majority of cases of pneumonia, and this is a potential topic for quality improvement projects.

### 4.3. Further Investigations and Additional Diagnoses

Chest CTs were also performed significantly less in the AP group than in the non-AP group. CTs were ordered following the diagnosis of pneumonia, when a different pathophysiology was also suspected, such as a pulmonary embolism or a tumour. As a result of the scans, not only were a clinically substantial number of cases (39.2%) found to have no detectable pneumonia but also more than 40% were diagnosed with an alternative condition. Many were life-limiting, such as a newly identified cancer (13.1%), new metastases of what was thought to be stable cancer (6.2%), and pulmonary embolism (10.8%). In other cases, conditions that called for further investigations or interventions, such as pleural effusion, pulmonary oedema, and pneumothoraces were detected. This compares to the results of a previous study, in which 27.3% of patients admitted for CAP had a non-pneumonia diagnosis upon discharge [45]. Considering the high ratio of an alternative diagnosis being identified, there may be more undiagnosed cases in patients who did not undergo a CT scan, especially in the AP group, which was significantly less investigated. A diagnosis of AP or being frail does not justify fewer investigations. Patients diagnosed with AP who are generally frailer and have more comorbidities are also at risk of these conditions. Additionally, many patients with AP had been previously admitted for pneumonia, which also raises suspicion of a different condition, such as a tumour, empyema, or tuberculosis. In the clinical setting, once AP is suspected, it is unfortunately not uncommon for a clinician’s mind to drift towards antibiotic treatment, NBM, and attaining a ‘do not resuscitate’ order, rather than logically rethinking their own clinical reasoning process and considering further investigation. If the history, physical examination, or other screening tests suggest the possibility of another condition, further investigation should be considered, including (but not limited to) CT scans.

### 4.4. Diagnosis of New Causes of Aspiration

Among the 803 cases, 35 cases (4.4%) were later diagnosed with a cause of aspiration during their hospital stay. These included life-threatening conditions, such as stroke or first-degree atrioventricular block, or treatable conditions, such as drug-induced aspirations. The non-AP group was less likely to be diagnosed with a cause of aspiration than the AP group (3.3% vs. 9.7%, respectively). This may, to some extent, be due to less investigation being performed to find a cause of aspiration in the non-AP group. If underlying causes of aspirations are left undiagnosed, it may lead to the progression of the condition, as well as recurrent pneumonia. It is difficult to accurately decide whether a patient has AP or non-AP with the limited information available at the initial patient contact. Therefore, when diagnosing older adults with pneumonia, it is essential to attain a detailed history of both acute and chronic symptoms with regard to possible aspiration, from the patient and family members or caregivers [46]. Rather than investigating the cause only in patients diagnosed with AP (of which the diagnosis may be inaccurate), it is recommended to consider the causes of aspiration in all pneumonia in older adults.

### 4.5. Management of the Patient

The choice of antimicrobial treatment differed between the two groups, understandably. The AP group was far more likely to be treated with the triple therapy antimicrobial regimen recommended for AP under the Trust guidelines. However, as few patients in the AP group underwent blood or sputum cultures, there is not enough information to confirm that this broad coverage was necessary. It may have put patients at risk of side effects, bacterial translocation, and future antimicrobial resistance. As the diagnosis of AP and non-AP remains ambiguous, deciding on antimicrobial treatment depending on these presumptive diagnoses entails clinical risk. Recently, it was recommended to start with narrow spectrum antimicrobials if the patient conditions allow and consider escalating the coverage if microbiology results are suggestive of resistant pathogens [17,44]. This is based on the increasing evidence suggesting a shift in causative pathogens of AP from anaerobic to aerobic organisms [47], and that anaerobic organisms causing AP are generally sensitive to narrow spectrum antimicrobials [15]. Therefore, it would be more clinically suitable if the initial antimicrobial treatment were to be selected by the patient’s condition, background, and previous microbiology results whilst conducting new microbial investigations, rather than deciding the treatment on the basis of a presumptive diagnosis.

Patients with a diagnosis of AP were also more likely to be made NBM initially, which is also not an ideal method of management. Not only will depriving elderly patients of food put them at high risk of malnutrition and delirium, but the disuse of the swallowing function will only make their swallowing worse. Studies in older patients with AP have reported that putting patients on NBM is an independent predictive factor for prolonged treatment and a greater decline in swallowing function [48], while another study on older patients with pneumonia has reported that the lack of energy intake during the first week of admission was an independent risk factor for mortality, recovery, and recurrence [49]. Therefore, unless a patient is at a high risk of choking, distress, and hypoxia (such as the patient being unconscious or in severe respiratory distress), it is recommended to assess their swallowing function and continue some sort of oral intake. It is common practice for patients to be kept NBM over the weekend due to there being no SLTs to assess the swallowing function, or for fear of their condition worsening. However, there has been a report stating that starting oral intake on weekends showed a better prognosis on patients with AP diagnosis than starting on weekdays [50]. Initiating oral intake early and using other methods of nutrition therapy, such as nasogastric feeding, is important in the prevention of malnutrition and sarcopenia [51].

When aspiration is suspected, early SLT assessment and intervention has been known to improve patient outcomes [52]. SLTs were only referred in 17.8% of patients diagnosed with non-AP. However, as the diagnoses of non-AP were not based on any swallow assessments, it is suspected that there are many patients with underdiagnosed dysphagia and aspiration in this group. Studies show that in the older population, 27% of those in the community [53] and 55% of those hospitalized, up to 85.9% of those with dementia [54], and 91% hospitalized with CAP [55] have swallowing disorders. By presumptively diagnosing a patient with non-AP, we may be depriving them of the opportunity to identify and treat their dysphagia and prevent further pneumonia.

### 4.6. Suggestions of a Paradigm Shift in the Diagnosis of Pneumonia

Accurately diagnosing a patient is the first step towards treating them effectively and preventing any further conditions. By making a presumptive diagnosis of AP, clinicians are potentially denying patients appropriate investigation and management. We may be exposing them to inappropriate antibiotics and unwanted dietary restrictions, putting them at risk of unnecessary side effects and malnutrition. Moreover, by tentatively ruling out AP, we may be neglecting the opportunities to investigate the swallowing functions or underlying causes of aspiration in these patients. 

**Figure 2:**
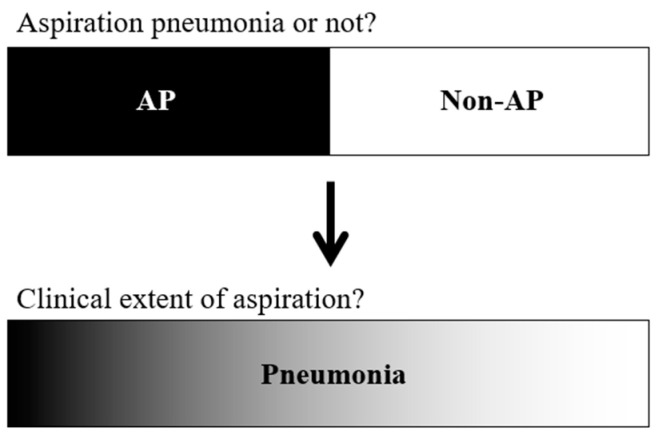
A paradigm shift in the diagnosis of pneumonia in older adults. Currently, the diagnosis of pneumonia in older adults is polarized among aspiration pneumonia (AP) or non-AP. We suggest the necessity of a paradigm shift in this process, where the diagnosis is pneumonia, and all older adults are assessed for the extent of clinical suspicion of aspiration.

AP is a highly commonly diagnosed condition in the elderly population, yet there is still no clear unified way to diagnose it. This conundrum, along with the results of our study, indicates that AP and non-AP are not straightforward black-or-white matters; rather, aspiration is an aetiology of pneumonia that can affect patients in different degrees. Therefore, when making a diagnosis of CAP, rather than concluding that a patient has AP or non-AP, it is more clinically relevant to explore to what extent aspiration contributes to pneumonia (Figure 2), along with other clinical aspects. We suggest this paradigm shift in the diagnosis of pneumonia in older persons. All patients with pneumonia should be considered for investigation according to the clinical appropriateness depending on their condition, rather than their ‘label’ of AP or non-AP. Clinicians should consider the clinical state of the patient, assess their ability to swallow and expectorate any aspiration, seek causative organisms, and investigate any underlying cause of dysphagia and aspiration or alternative diagnoses, as previously suggested [46]. To assess the extent of aspiration, simple individual screenings, such as the water swallow test or simple swallow provocation test, could be carried out, or mealtime assessments may be of use [56]. Depending on the screening results and availability, further bedside or instrumental assessment may be considered. There are also tools developed to guide the clinical management of pneumonia in older patients with a focus on AP, such as the Assessment of Swallowing Ability for Pneumonia (ASAP) [57] and the aspiration pneumonia cause investigation algorithm [58]. In pneumonia in older adults, rather than relying on a largely heterogeneous diagnostic term, the optimal care may be to manage patients according to attentive bedside examination and holistic assessment.

### 4.7. Strengths and Weaknesses of This Study

There are some limitations associated with this study. First, this was a single centre, retrospective study, performed in 2021, where the effects of the COVID-19 pandemic may still have some effect on the patient population. The situation may differ in other settings. However, this was a fairly large study involving an initial list of over 1400 patients with pneumonia, from a 521-bed acute hospital. To our knowledge, there have not been many studies undertaken on older adults diagnosed with AP in the UK, and this study is important to highlight current practices in the area. Second, diagnoses were extracted from medical records. Results may have been affected by how the diagnoses were made and documented. However, as the purpose of this study was to investigate what clinical factors appear to influence the diagnoses of AP and non-AP in the daily clinical setting, this was considered the feasible and optimal method for a retrospective study. We believe this is a meaningful step towards contemplating the challenging conundrum of diagnosing AP.

## 5. Conclusions

In conclusion, older patients admitted for CAP were more likely to be diagnosed with AP if they were frailer and had more underlying conditions. The diagnosis of AP seemed to have led to the choice for broader antimicrobial coverage, NBM, and SLT referrals, rather than an assessment of the microbial risk factors or swallowing function. This study highlights the potential risks of a presumptive diagnosis and suggests a shift towards a more careful assessment of each patient’s condition.

## Figures and Tables

**Table 1:** Patient background and past medical history.

Factor	AP (*n* = 134)		Non-AP (*n* = 669)		*p*-Value
Background	*n*	%, IQR	*n*	%, IQR	
Male (*n*, %)	72	(53.7)	351	(52.5)	0.79
Age (median, IQR)	85	(80–90)	84	(80–89)	0.11
Care home/nursing home (*n*, %)	40	(29.9)	76	(11.4)	<0.001
Clinical frailty scale (median, IQR)	6	(5–7)	5	(4–6)	<0.001
SARC-F score (median, IQR)	7	(4–10)	4	(2–7)	<0.001
**Past medical history, comorbidities**					
Stroke (*n*, %)	28	(20.9)	102	(15.2)	0.11
Neurologic disorder (*n*, %)	23	(17.2)	28	(4.2)	<0.001
Dementia (*n*, %)	69	(51.5)	154	(23.0)	<0.001
Other mental disorder (*n*, %)	15	(11.2)	69	(10.3)	0.76
Gastroesophageal reflux disease (*n*, %)	8	(6.0)	28	(4.2)	0.36
Other gastroesophageal disorder (*n*, %)	17	(12.7)	53	(7.9)	0.07
Ischemic/congestive cardiac condition (*n*, %)	31	(23.1)	207	(30.9)	0.07
Type 2 diabetes mellitus (*n*, %)	22	(16.4)	161	(24.1)	0.05
Chronic respiratory disorder (*n*, %)	22	(16.4)	198	(29.6)	<0.05
Active cancer (*n*, %)	18	(13.4)	93	(13.9)	0.89
Head and neck cancer (*n*, %)	2	(1.5)	7	(1.0)	0.65
Immunodeficiency (*n*, %)	5	(3.7)	72	(10.8)	<0.05
Pneumonia within 1 year (*n*, %)	38	(28.4)	135	(20.2)	<0.05
Number of daily drugs (median, IQR)	6	(5–9)	7	(4–9)	0.13
Known dysphagia (*n*, %)	60	(44.8)	40	(6.0)	<0.001
**Risk factors of multi-drug resistant pathogens**					
Hospital admission ≥2 days in the past 90 days (*n*, %)	34	(25.4)	184	(27.5)	<0.001
Haemodialysis (*n*, %)	2	(1.5)	5	(0.7)	0.33
Intravenous antibiotic therapy in the last 90 days (*n*, %)	26	(19.4)	122	(18.2)	0.75

(AP: aspiration pneumonia, IQR: interquartile range).

**Table 2:** Presenting condition.

Factor	AP (*n* = 134)		Non-AP (*n* = 669)		*p*-Value
Symptoms	*n*	%, IQR	*n*	%, IQR	
Cough (*n*, %)	52	(38.8)	314	(46.9)	0.08
Purulent sputum (*n*, %)	30	(22.4)	166	(24.8)	0.55
Pleuritic pain (*n*, %)	1	(0.7)	33	(4.9)	<0.05
Dyspnoea (*n*, %)	40	(29.9)	380	(56.8)	<0.001
Fever (*n*, %)	27	(20.1)	191	(28.6)	<0.05
Coughing on oral intake (*n*, %)	32	(23.9)	12	(1.8)	<0.001
Vomiting (*n*, %)	60	(44.8)	43	(6.4)	<0.001
Altered mental status from baseline (*n*, %)	43	(32.1)	150	(22.4)	<0.05
**Severity of the pneumonia**					
CURB-65, median (*n*, IQR)	2	(2–3)	2	(1–2)	<0.001
Pneumonia severity index (median, IQR)	107	(95–128)	103	(84–119)	<0.001

(AP: aspiration pneumonia, IQR: interquartile range).

**Table 3:** Management following the diagnosis of pneumonia.

Factor	AP (*n* = 134)		Non-AP (*n* = 669)		*p*-Value
Further Investigations Performed	*n*	%	*n*	%	
Blood culture (*n*, %)	36	(26.9)	252	(37.7)	<0.05
Sputum culture (*n*, %)	6	(4.5)	40	(6.0)	0.49
Urine *S. pneumoniae* antigen (*n*, %)	0	(0)	11	(1.6)	0.23
Urine *Legionella* antigen (*n*, %)	2	(1.5)	46	(6.9)	<0.05
Chest CT scan (*n*, %)	12	(9.9)	118	(17.6)	<0.05
**Antimicrobial treatment**					
AP triple therapy (*n*, %)	71	(53.0)	19	(2.8)	<0.001
**Actions on admission**					
SLT referral (*n*, %)	94	(70.1)	119	(17.8)	<0.001
Nil by mouth orders (*n*, %)	70	(52.2)	49	(7.3)	<0.001
VFSS/FEES (*n*, %)	4	(3.0)	3	(0.4)	<0.05

(AP: aspiration pneumonia, IQR: interquartile range, CT: computed tomography. SLT: speech and language therapist, VFSS: videofluoroscopic swallow study, FEES: fibreoptic endoscopic evaluation of swallowing).

**Table 4:** Chest CT findings.

Findings	AP (*n* = 12)		Non-AP (*n* = 118)		Total (*n* = 130)	
	*n*	%	*n*	%	*n*	%
No pneumonia	4	(33.3)	47	(39.8)	51	(39.2)
Only pneumonia	6	(50.0)	56	(47.5)	62	(47.7)
Other diagnosis (+/− pneumonia)	5	(41.7)	51	(43.2)	56	(43.1)
Pulmonary embolism	0	(0)	14	(11.9)	14	(10.8)
Cancer, previously unidentified	1	(8.3)	16	(13.6)	17	(13.1)
Lung	1	(8.3)	12	(10.2)	13	(10.0)
Other (mediastinal, breast, liver, adrenal)	0	(0)	4	(3.4)	4	(3.1)
New lung metastasis of known cancer	2	(16.7)	6	(5.1)	8	(6.2)
New lung nodules (no pathological diagnosis)	0	(0)	3	(2.5)	3	(2.3)
Pleural effusion	0	(0)	6	(5.1)	6	(4.6)
Pulmonary oedema	0	(0)	3	(2.5)	3	(2.3)
Other (ILD, pneumothorax, emphysema, hiatal hernia)	2	(16.7)	3	(2.5)	5	(3.8)

(AP: aspiration pneumonia, ILD: interstitial lung disease).

**Table 5:** Newly diagnosed causes of aspiration.

Causes	Total (*n* = 35)	
	*n*	(%)
**N** **eurologic**	13	(37.1)
Stroke	7	(20.0)
Dementia	5	(14.3)
Bell’s palsy	1	(2.9)
**Head and neck**	3	(8.6)
Oral thrush	2	(5.7)
Laryngocele	1	(2.9)
**Cardiopulmonary**	3	(8.6)
First-degree atrioventricular block, syncope	1	(2.9)
Chronic obstructive lung disease	1	(2.9)
Obstructive sleep apnoea	1	(2.9)
**Gastrointestinal**	10	(28.6)
Hiatal hernia	4	(11.4)
Cholecystitis	1	(2.9)
Metastatic oesophageal obstruction	2	(5.7)
Oesophageal stenosis	1	(2.9)
Candida esophagitis	1	(2.9)
Achalasia	1	(2.9)
**Drug induced**	6	(17.1)
Hypercalcemia (osteoporosis treatment)	2	(5.7)
Hypo-delirium (antipsychotic, antidepressant)	2	(5.7)
Opioid toxicity	1	(2.9)
Nausea (iron supplement)	1	(2.9)

## Data Availability

The data presented in this study are available upon request from the corresponding author. The data are not publicly available due to privacy and ethical reasons.

## Appendix A

**Table A1:** Patient background and past medical history, excluding patients without pneumonia, findings on CT.

Factor	AP (*n* = 130)		Non-AP (*n* = 622)		*p*-Value
Background	*n*	%, IQR	*n*	%, IQR	
Male (*n*, %)	70	(53.8)	326	(52.4)	0.76
Age (median, IQR)	85	(80–90)	84	(80–89)	0.11
Care home/nursing home (*n*, %)	40	(30.8)	72	(11.6)	<0.001
Clinical frailty scale (median, IQR)	6	(5–7)	5	(4–6)	<0.001
SARC-F score (median, IQR)	7	(4–10)	4	(2–7)	<0.001
**Past medical history, comorbidities**					
Stroke (*n*, %)	28	(21.5)	99	(15.9)	0.12
Neurologic disorder (*n*, %)	22	(16.9)	28	(4.5)	<0.001
Dementia (*n*, %)	68	(52.3)	147	(23.6)	<0.001
Other mental disorder (*n*, %)	15	(11.5)	63	(10.1)	0.63
Gastroesophageal reflux disease (*n*, %)	8	(6.2)	28	(4.5)	0.42
Other gastroesophageal disorder (*n*, %)	17	(13.1)	52	(8.4)	0.09
Ischemic/congestive cardiac condition (*n*, %)	29	(22.3)	198	(31.8)	<0.05
Type 2 diabetes mellitus (*n*, %)	21	(16.2)	152	(24.4)	<0.05
Chronic respiratory disorder (*n*, %)	21	(16.2)	184	(29.6)	<0.05
Active cancer (*n*, %)	17	(13.1)	85	(13.7)	0.86
Head and neck cancer (*n*, %)	2	(1.5)	6	(1.0)	0.63
Immunodeficiency (*n*, %)	5	(3.8)	66	(10.6)	<0.05
Pneumonia within 1 year (*n*, %)	36	(27.7)	126	(20.3)	0.06
Number of daily drugs (median, IQR)	6	(5–9)	7	(4–9)	0.07
Known dysphagia (*n*, %)	60	(46.2)	40	(6.4)	<0.001
**Risk factors of multi-drug resistant pathogens**					
Hospital admission ≥2 days in the past 90 days (*n*, %)	33	(25.4)	173	(27.8)	<0.001
Haemodialysis (*n*, %)	2	(1.5)	5	(0.8)	0.35
Intravenous antibiotic therapy in the last 90 days (*n*, %)	25	(19.2)	113	(18.2)	0.78

(AP: aspiration pneumonia, IQR: interquartile range).

**Table A2:** Presenting condition, excluding patients without pneumonia findings on CT.

Factor	AP (*n* = 130)		Non-AP (*n* = 622)		*p*-Value
Symptoms	*n*	%, IQR	*n*	%, IQR	
Cough (*n*, %)	51	(39.2)	293	(47.1)	0.08
Purulent sputum (*n*, %)	30	(23.1)	151	(24.3)	0.55
Pleuritic pain (*n*, %)	1	(0.8)	28	(4.5)	<0.05
Dyspnoea (*n*, %)	40	(30.8)	350	(56.3)	<0.001
Fever (*n*, %)	26	(20.0)	181	(29.1)	<0.05
Coughing on oral intake (*n*, %)	31	(23.8)	12	(1.9)	<0.001
Vomiting (*n*, %)	57	(43.8)	42	(6.8)	<0.001
Altered mental status from baseline (*n*, %)	43	(33.1)	144	(23.2)	<0.05
**Severity of the pneumonia**					
CURB-65, median (*n*, IQR)	2	(2–3)	2	(1–2)	<0.001
Pneumonia severity index (median, IQR)	107	(95–128)	103	(85–119)	<0.05

(AP: aspiration pneumonia, IQR: interquartile range).
